# Care Strategies in Emergency Management of Acute Ischemic Stroke: A Systematic Review

**DOI:** 10.3390/neurolint18080148

**Published:** 2026-08-07

**Authors:** Paula Sánchez-Fernandez, Álvaro Astasio-Picado, Jesús Jurado-Palomo

**Affiliations:** 1 Faculty of Health Sciences, University of Castilla-La Mancha, 45600 Toledo, Spain; paula.sanchez29@alu.uclm.es; 2 Physiotherapy, Nursing and Physiology Department, Faculty of Health Sciences, University of Castilla-La Mancha, 45600 Toledo, Spain; jesus.juradopalomo@uclm.es; 3 Intensive Care Unit, Hospital Virgen del Puerto, 10600 Plasencia, Spain; 4 Faculty of Economics and Business, Universitat Oberta de Catalunya, 09018 Barcelona, Spain

**Keywords:** acute ischemic stroke, emergency department, multidisciplinary care, treatment delay, clinical outcomes

## Abstract

Introduction: Stroke remains a leading cause of mortality and long-term disability worldwide. Coordinated care delivered through protocol-driven multidisciplinary teams enables rapid diagnosis and treatment, both of which are essential for improving clinical outcomes. Objective: Our objective was to analyze care strategies in the emergency management of acute ischemic stroke, focusing on treatment times and influencing factors, interventions implemented in emergency settings and their impact on clinical outcomes, and the contribution of healthcare professionals to optimizing stroke care. Methods: A systematic review was conducted in accordance with the PRISMA 2020 Statement. Current evidence published between 2021 and 2026 was retrieved from five databases: PubMed, Web of Science, Scopus, Cochrane Library, and SciELO. Eligibility criteria were applied, and methodological quality and risk of bias were assessed using validated appraisal tools. Results: Fourteen studies involving a total of 6384 patients were included. Factors such as healthcare system reorganization, multidisciplinary teamwork, early protocol activation, and the use of stroke-specific triage scales significantly influenced treatment times. Coordinated and protocolized care pathways, together with evidence-based clinical interventions, were consistently associated with higher thrombolysis rates and shorter treatment times, while improvements in neurological and functional recovery were reported in several, but not all, studies. The integration of specialized stroke professionals and structured emergency care processes contributed to reducing treatment delays and improving patient outcomes. Conclusions: Critical treatment times in acute ischemic stroke are influenced by multiple factors, and targeted organizational and clinical strategies can accelerate diagnosis and treatment. Although improvements in clinical outcomes were not consistently demonstrated across all included studies, the available evidence consistently supports protocol-driven multidisciplinary emergency care as an effective strategy for reducing treatment delays and optimizing acute ischemic stroke management.

## 1. Introduction

Stroke remains one of the most significant global health challenges, representing a leading cause of mortality and long-term disability worldwide. The burden of stroke extends beyond its clinical consequences, generating substantial social and economic costs for patients, families, healthcare systems, and society as a whole. Furthermore, demographic changes, particularly population aging and the increasing prevalence of cardiovascular risk factors, are expected to contribute to a further rise in stroke incidence and prevalence over the coming decades [1].

Stroke is defined as the sudden onset of a focal neurological deficit resulting from a vascular disturbance affecting cerebral circulation. This disruption may occur due to a reduction in cerebral blood flow, known as ischemia, or as a consequence of intracranial hemorrhage. Both mechanisms lead to cerebral hypoxia and can result in irreversible neurological damage if appropriate diagnostic and therapeutic interventions are not implemented promptly [2]. As a consequence, stroke is considered a medical emergency that requires immediate recognition and management to minimize brain injury and improve patient outcomes.

Among all cerebrovascular diseases, ischemic stroke is the most frequent subtype, accounting for approximately 80–85% of all stroke cases, whereas hemorrhagic stroke represents the remaining 15–20% [3,4,5,6,7,8]. Ischemic stroke occurs when blood flow to a region of the brain is interrupted by arterial occlusion, leading to tissue ischemia and infarction. Importantly, this subtype is potentially amenable to reperfusion therapies, including intravenous thrombolysis and mechanical thrombectomy, which have substantially transformed acute stroke management during recent decades [4]. In contrast, hemorrhagic stroke results from the rupture of a cerebral blood vessel and requires different therapeutic approaches [4].

Stroke continues to be a major cause of death and disability in Spain, where it is the leading cause of mortality among women and the second leading cause among men [3]. Its occurrence is strongly associated with several modifiable and non-modifiable risk factors. Among the most relevant are hypertension, diabetes mellitus, obesity, dyslipidemia, and advanced age. Environmental factors, such as air pollution and rising ambient temperatures, have also been linked to increased stroke risk. Additionally, behavioral factors, including tobacco consumption, physical inactivity, excessive alcohol intake, and unhealthy dietary habits, contribute significantly to the global burden of cerebrovascular disease [9,10].

Over the last few decades, growing evidence has demonstrated that early recognition of stroke symptoms and rapid implementation of evidence-based interventions are crucial determinants of prognosis. Delays in diagnosis and treatment are associated with increased neuronal loss, larger infarct volumes, poorer functional outcomes, and higher mortality rates [3]. The widely accepted concept that “time is brain” reflects the urgency of acute stroke management, emphasizing that every minute of persistent cerebral ischemia results in irreversible neuronal damage and reduced opportunities for functional recovery.

To address this time-sensitive condition, many healthcare systems have developed structured pathways aimed at accelerating diagnosis and treatment. In Spain, acute stroke management is organized through the “Código Ictus” (Stroke Code), a protocol-based emergency response system designed to ensure rapid identification, prioritization, transportation, diagnosis, and treatment of eligible patients [3,4,5,6,7,8,11]. This strategy involves close coordination among prehospital emergency services, emergency departments, stroke units, radiology departments, neurologists, and other healthcare professionals participating in the care continuum.

The effectiveness of acute stroke care depends not only on the availability of reperfusion therapies but also on the efficiency of organizational processes. Previous studies have shown that multidisciplinary collaboration, early activation of stroke protocols, streamlined diagnostic pathways, and optimized in-hospital workflows contribute significantly to reducing critical treatment times, including door-to-imaging and door-to-needle intervals [3]. The implementation of standardized clinical pathways has also been associated with increased rates of thrombolytic treatment, shorter hospital stays, and improved neurological and functional outcomes.

Healthcare professionals play a central role throughout the acute stroke pathway. Their responsibilities include the early identification of stroke symptoms, triage prioritization, activation of emergency protocols, clinical monitoring, implementation of therapeutic interventions, and prevention of complications. Effective communication and coordination among multidisciplinary team members are essential to ensure timely and evidence-based care delivery [6,7,8,12]. Consequently, understanding the organizational and clinical strategies that facilitate rapid stroke management has become a priority for improving healthcare quality and patient outcomes.

Given the strong association between treatment times and prognosis, there is a growing need to identify and evaluate the factors that influence the efficiency of acute ischemic stroke care. Therefore, this systematic review aims to examine the current evidence regarding care strategies implemented in emergency settings, the determinants of treatment delays, and the impact of organizational and clinical interventions on the outcomes of patients with acute ischemic stroke [13].

The general objective of this study was to analyze the available scientific evidence on care strategies implemented in the emergency management of patients with acute ischemic stroke.

Unlike previous reviews focused on individual components of acute stroke care, the present systematic review synthesizes the most recent evidence (2021–2026) regarding organizational, multidisciplinary, and protocol-based emergency care strategies. By integrating evidence from different healthcare settings, this review aims to provide a comprehensive overview of factors influencing treatment efficiency and patient outcomes in acute ischemic stroke.

## 2. Materials and Methods

### 2.1. Study Design

This study was conducted as a systematic review of the scientific literature with the aim of synthesizing the current evidence regarding care strategies implemented in the emergency management of acute ischemic stroke. The review was developed in accordance with the Preferred Reporting Items for Systematic Reviews and Meta-Analyses (PRISMA 2020) Statement, which provides a standardized framework to ensure methodological rigor, transparency, and reproducibility in systematic reviews. The PRISMA 2020 guidelines comprise a 27-item checklist designed to improve the quality of reporting and facilitate evidence-based decision-making in healthcare [14].

To enhance methodological transparency and reduce the risk of reporting bias, the review protocol was prospectively registered in the International Prospective Register of Systematic Reviews (PROSPERO) under registration number CRD420261335376.

### 2.2. Eligibility Criteria

Eligibility criteria were established before the literature search and applied consistently throughout the study selection process. Studies published between January 2021 and February 2026 were considered eligible in order to capture the most recent evidence regarding acute ischemic stroke care. Only publications written in English or Spanish were included.

The review considered studies directly related to the emergency management of acute ischemic stroke, including randomized controlled trials, non-randomized intervention studies, systematic reviews, and meta-analyses. Particular attention was given to studies addressing treatment times, organizational strategies, multidisciplinary care pathways, clinical interventions, and patient outcomes. To ensure the inclusion of robust evidence, only studies published in peer-reviewed journals with recognized scientific quality were considered.

Studies published before 2021, articles unrelated to the research topic, publications in languages other than English or Spanish, narrative reviews, editorials, commentaries, conference abstracts, and studies presenting insufficient methodological quality were excluded from the review.

### 2.3. Research Question

The research question was developed according to the Population, Intervention, Comparison, Outcome (PICO) framework. The population of interest consisted of adult patients diagnosed with acute ischemic stroke. The intervention included structured and protocol-driven emergency care strategies implemented within stroke care pathways, including multidisciplinary interventions aimed at reducing treatment delays and optimizing patient management. The comparison involved conventional care or the absence of structured stroke protocols when applicable. The outcomes of interest included treatment times, clinical and functional outcomes, and the occurrence of complications.

Accordingly, the review sought to answer the following question: *In adult patients with acute ischemic stroke, what is the impact of protocolized emergency care strategies on treatment times, clinical outcomes, and complications compared with standard care?*

### 2.4. Information Sources

A comprehensive literature search was conducted in five major electronic databases: PubMed, Web of Science, Scopus, Cochrane Library, and SciELO. These databases were selected because of their extensive coverage of biomedical, clinical, and health sciences literature, as well as their relevance for stroke research and emergency care.

The search process was carried out between December 2025 and February 2026. Additionally, the reference lists of relevant articles were manually screened to identify potentially eligible studies that may not have been retrieved through the electronic database search.

### 2.5. Search Strategy

The search strategy was developed using a combination of controlled vocabulary terms derived from Medical Subject Headings (MeSH) and Health Sciences Descriptors (DeCS), together with free-text keywords. Boolean operators such as AND and OR were employed to maximize search sensitivity while maintaining specificity.

The principal search equations included combinations of terms related to stroke, emergency care, treatment delays, triage processes, and healthcare professionals involved in acute stroke management. Examples of the search strategies applied were:

“Stroke” AND (“emergency nurs” OR “triage nurs*”)*

*and*

*“Stroke” AND “time-to-treatment” AND nurs.*

The search strategy was developed using a combination of controlled vocabulary (MeSH and DeCS terms) and free-text keywords related to acute ischemic stroke, emergency management, stroke pathways, multidisciplinary care, treatment times, reperfusion therapies, triage, and healthcare professionals involved in stroke care. Boolean operators (AND/OR) were adapted to each database to maximize sensitivity while maintaining specificity.

Because the review aimed to identify organizational strategies implemented during emergency stroke care, broad searches initially retrieved a large number of records. Successive screening based on predefined eligibility criteria, methodological quality, relevance to the research question, and duplication removal substantially reduced the number of studies included in the qualitative synthesis. Database-specific search equations were adapted according to indexing systems, and manual screening of reference lists was additionally performed to identify potentially eligible studies.

### 2.6. Study Selection and Data Synthesis

The study selection process was conducted between December 2025 and February 2026. Following database searches, all retrieved records were exported and screened for duplicate publications. Subsequently, titles and abstracts were independently reviewed to identify studies potentially meeting the predefined eligibility criteria.

Articles considered relevant proceeded to full-text assessment. During this stage, studies were evaluated according to their methodological quality, relevance to the research question, and consistency with the defined population, interventions, and outcomes. Any disagreements regarding study eligibility were resolved through discussion among the reviewers until consensus was achieved.

Data extraction was performed by one reviewer using a standardized extraction form, while a second reviewer verified the accuracy and consistency of the collected information. Extracted variables included study design, country, sample characteristics, interventions, organizational strategies, treatment times, clinical outcomes, and key findings.

Due to the heterogeneity observed among study designs, interventions, and outcome measures, a narrative synthesis of the evidence was undertaken. Findings were grouped according to common themes, including factors influencing treatment times, organizational and multidisciplinary interventions, and their impact on patient outcomes.

Although the included studies differed in geographical setting and study design, they shared a common objective of evaluating organizational or clinical strategies aimed at optimizing emergency management of acute ischemic stroke. This conceptual consistency justified their inclusion in the qualitative synthesis despite methodological heterogeneity.

### 2.7. Methodological Quality Assessment and Risk of Bias

The methodological quality of the included studies was assessed using validated tools appropriate for each study design. Systematic reviews and meta-analyses were evaluated using *A Measurement Tool to Assess Systematic Reviews 2* (AMSTAR 2), which assesses methodological quality across sixteen domains and provides an overall appraisal of confidence in the review findings.

Randomized controlled trials were assessed using the Jadad Scale, which evaluates methodological rigor based on randomization procedures, blinding methods, and participant withdrawals. Non-randomized studies were appraised using the Newcastle–Ottawa Scale, which examines participant selection, comparability of study groups, and outcome assessment.

Risk of bias was also evaluated using design-specific instruments. Systematic reviews and meta-analyses were assessed with the *Risk of Bias in Systematic Reviews* (ROBIS) tool, which evaluates concerns across four domains related to eligibility criteria, study identification and selection, data collection and appraisal, and synthesis of findings [15].

For randomized controlled trials, the *Risk of Bias 2* (RoB 2) tool was employed. This instrument assesses potential bias arising from the randomization process, deviations from intended interventions, missing outcome data, outcome measurement, and selective reporting. Studies were classified as presenting low risk of bias, some concerns, or high risk of bias [16].

Non-randomized studies were evaluated using the *Risk Of Bias In Non-randomized Studies of Interventions* (ROBINS-I) tool. This framework examines bias related to confounding, participant selection, intervention classification, deviations from intended interventions, missing data, outcome measurement, and selective reporting. Overall risk of bias was categorized as low, moderate, serious, critical, or no information available [17].

## 3. Results

### 3.1. Study Selection Results

The literature search identified a substantial number of potentially relevant studies across the selected databases. After the removal of duplicate records and the application of predefined eligibility criteria, 14 studies were included in the final qualitative synthesis. These studies collectively involved 6384 patients and provided evidence regarding organizational strategies, multidisciplinary interventions, treatment times, and clinical outcomes in the emergency management of acute ischemic stroke.

**Figure 1:**
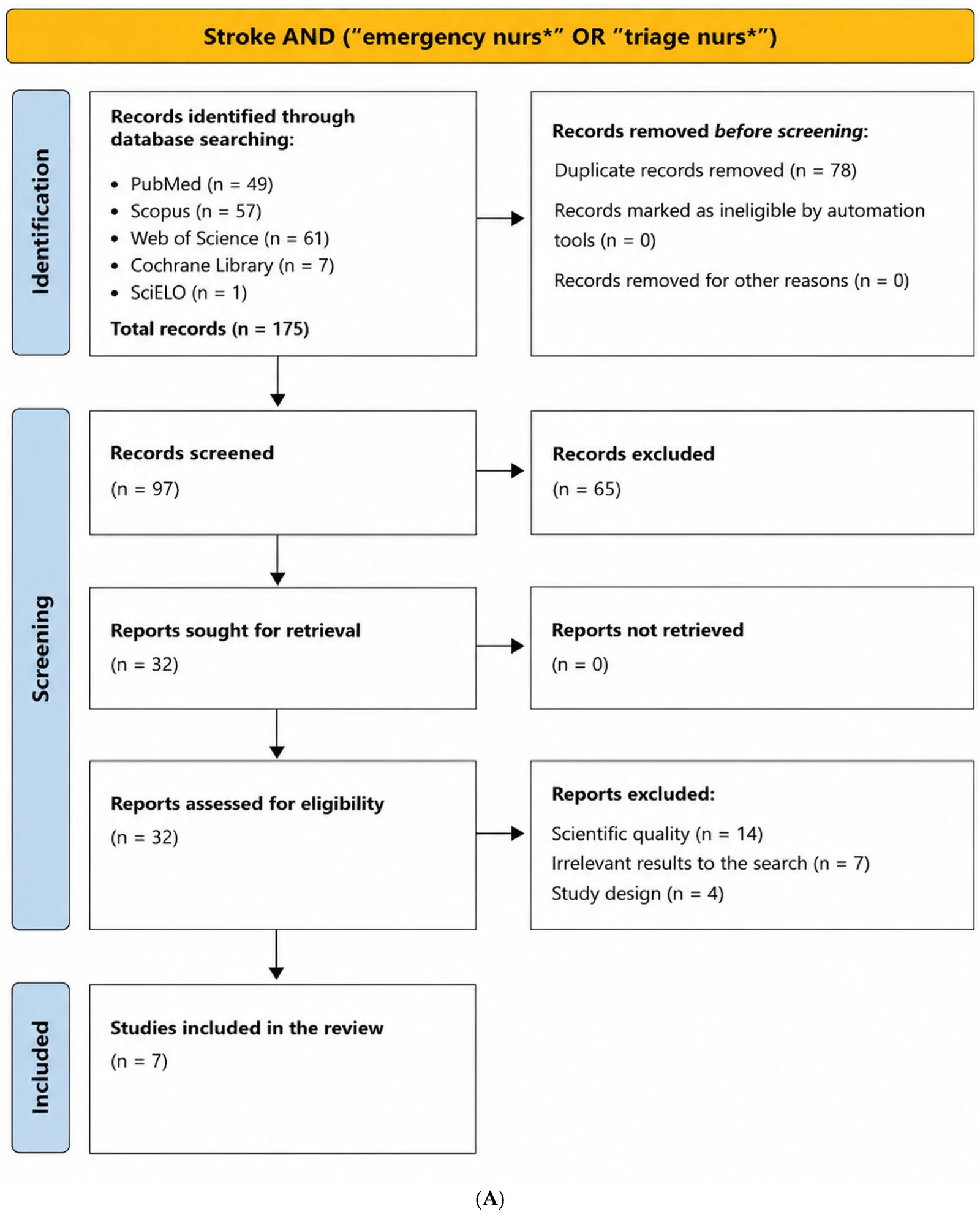

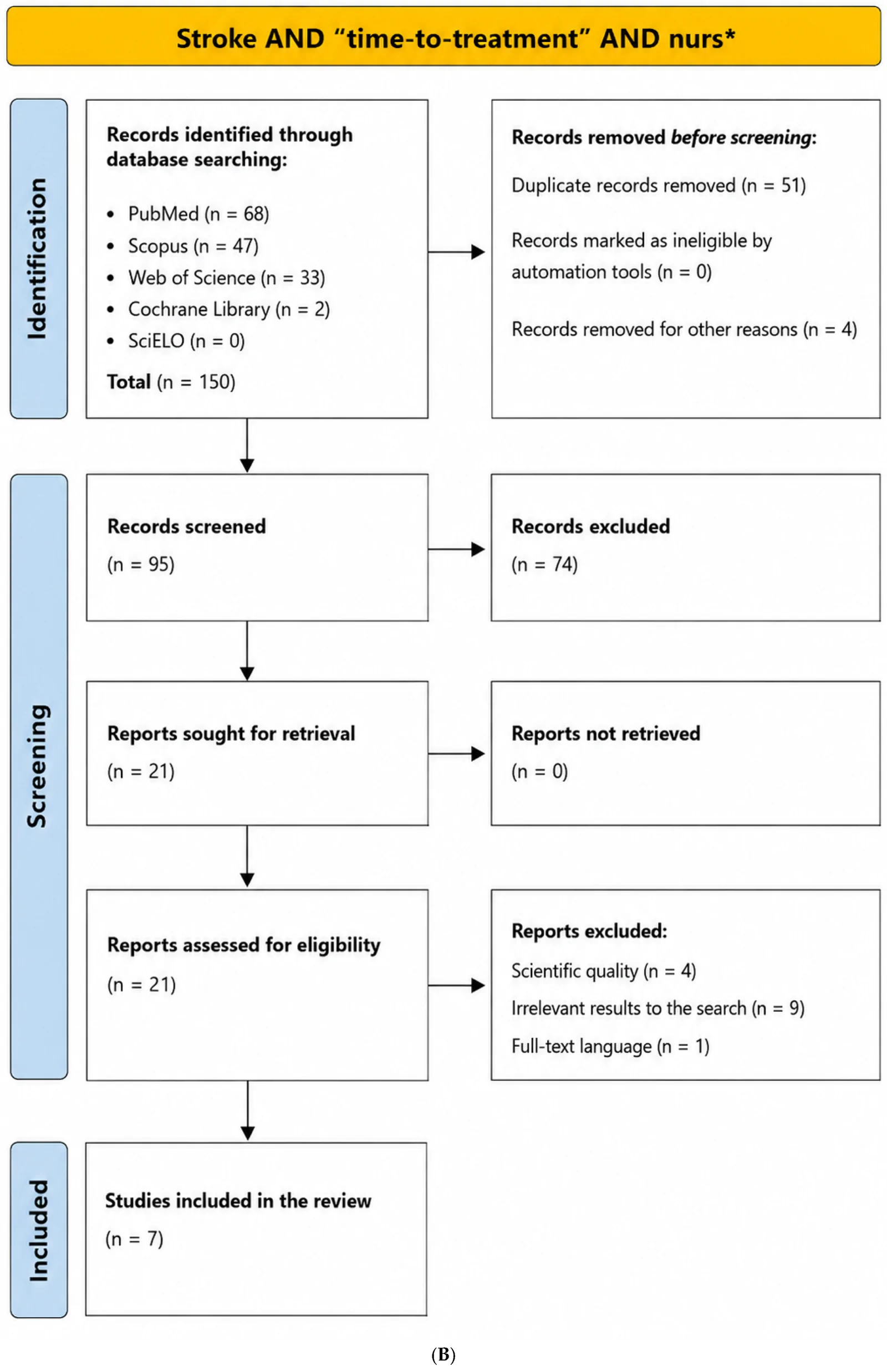
(**A**,**B**): Flowchart of the process of searching and selecting articles according to the PRISMA 2020 statement. Source: Prepared by the authors using the PrismaFlow Studio tool [18].

The study selection process was conducted according to the PRISMA 2020 guidelines and is presented in the PRISMA flow diagram (Figure 1A,B). Initial screening of titles and abstracts resulted in the exclusion of studies that did not meet the inclusion criteria. Subsequently, full-text assessment was performed to evaluate methodological quality, relevance to the research question, and consistency with the predefined population, intervention, and outcome criteria. Only studies fulfilling all eligibility requirements were included in the final review (Supplementary Table S1).

### 3.2. Study Characteristics

The main characteristics of the included studies are summarized in Appendices Table A1 and Table A2 (Appendix A and Appendix E), which presents information regarding authorship, year of publication, study objectives, design, sample characteristics, and methodological quality scores. The selected literature comprised randomized controlled trials, retrospective and prospective observational studies, systematic reviews, and meta-analyses, reflecting the diversity of evidence currently available on acute ischemic stroke care.

**Figure A1:**
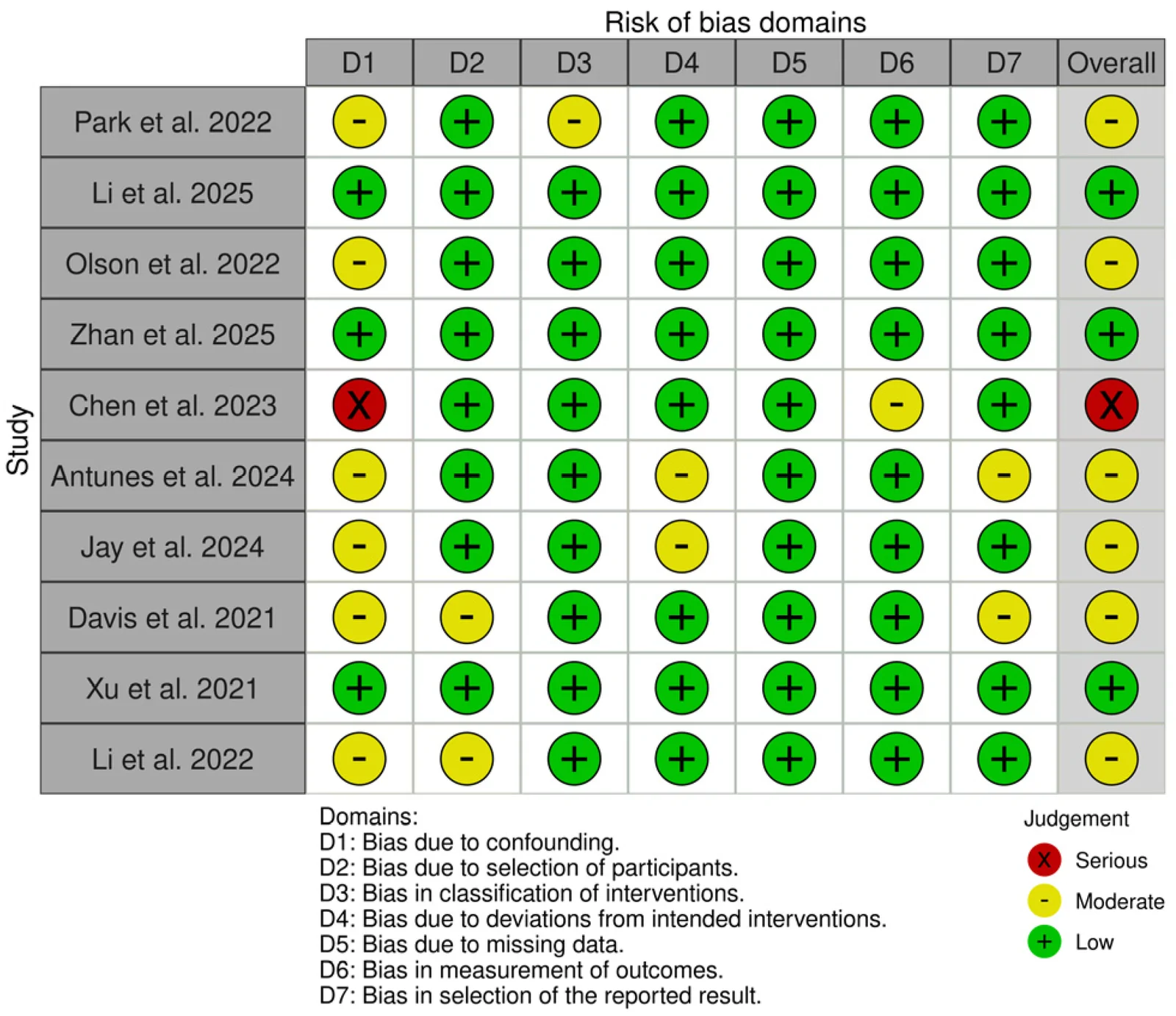
Assessment of bias risk using the ROBINS-I scale [19,20,21,22,23,24,28,29,31,32]. Source: Prepared by the authors using the Rovbis tool [34].

**Figure A2:**
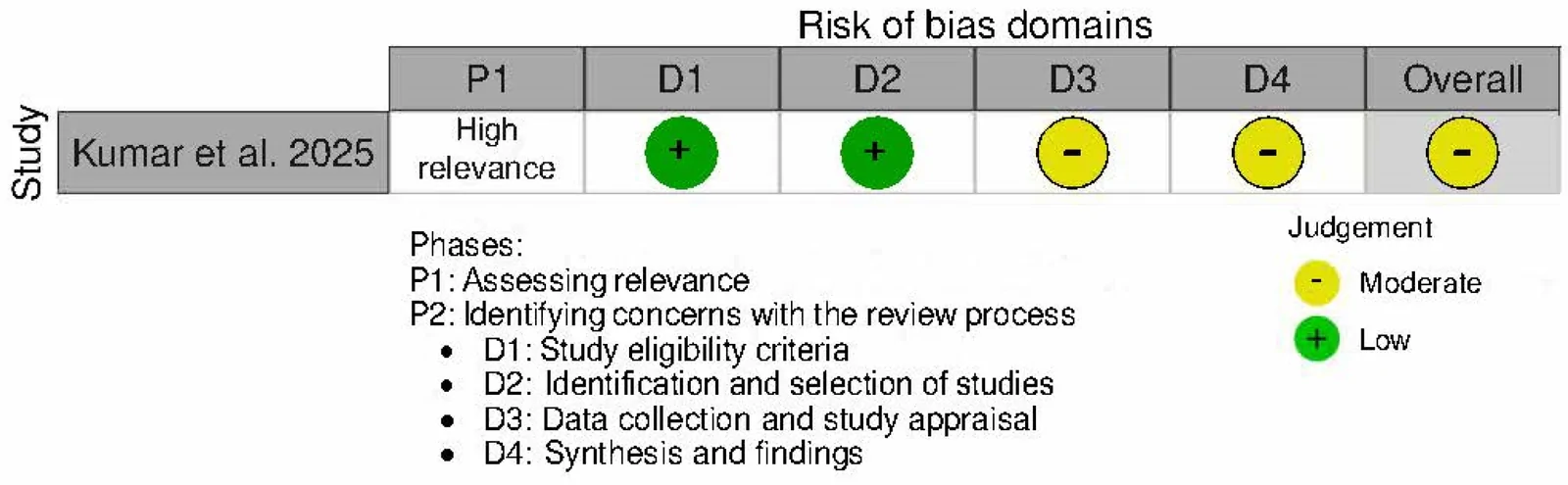
Assessment of bias risk using the ROBIS scale [30]. Source: Prepared by the authors using the ROBIS tool [34].

**Figure A3:**
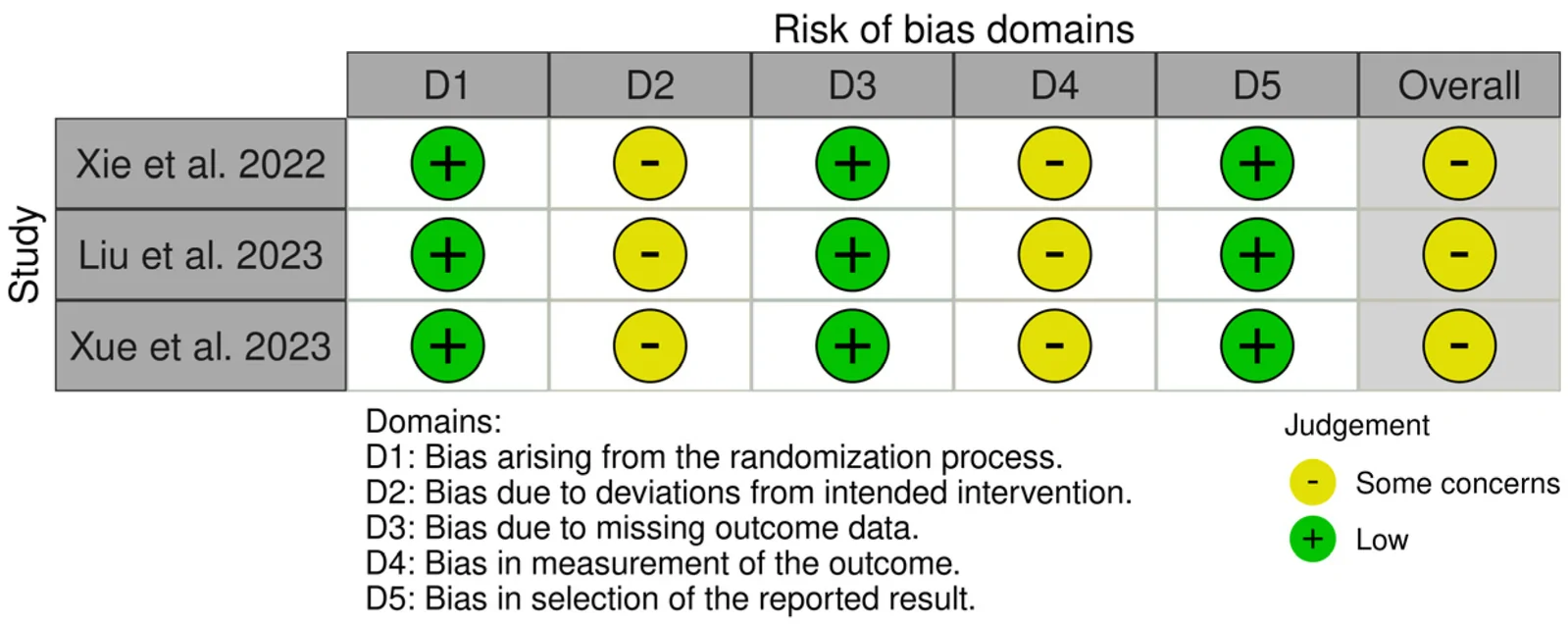
Assessment of the risk of bias using the RoB 2 scale [25,26,27]. Source: Prepared by the authors using the Rovbis tool [34].

The studies were conducted in different healthcare settings and countries, providing a broad perspective on organizational models, emergency department workflows, multidisciplinary approaches, and specialized stroke care pathways. Methodological quality was assessed using validated appraisal tools according to study design, while the risk of bias assessments are presented in Appendices Figure A1, Figure A2 and Figure A3.

### 3.3. Results of the Evidence Synthesis

#### 3.3.1. Factors Influencing Critical Treatment Times

**Figure 2:**
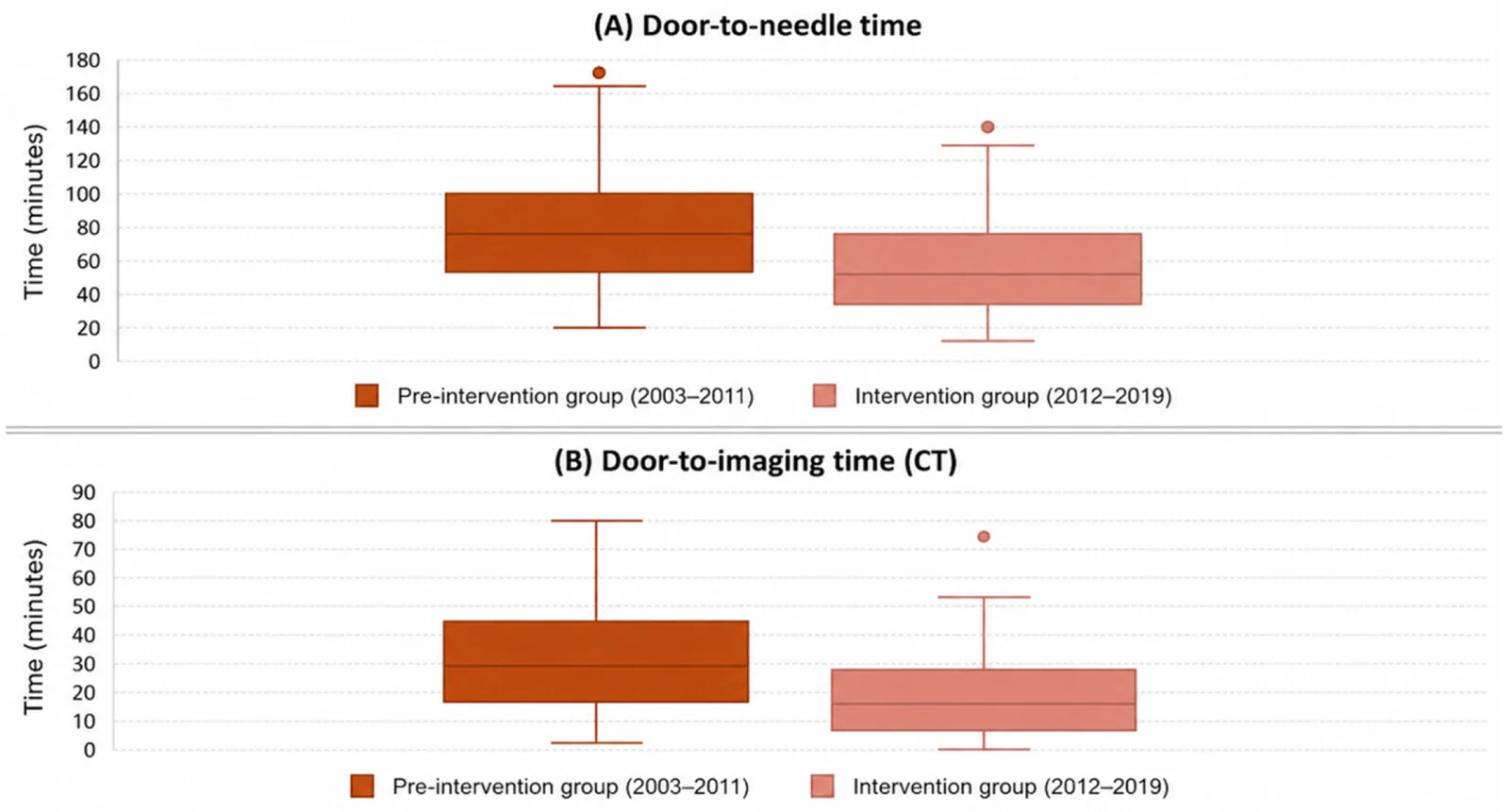
Evolution over the 17 years observed of door-to-needle (**A**) and door-to-CT (**B**) times. Source: Prepared by the authors using data from Park et al. [19].

Several studies identified organizational and clinical factors associated with improvements in critical treatment intervals during acute ischemic stroke management. One of the most comprehensive analyses was conducted by Park et al. [19], who evaluated the impact of multiple quality-improvement interventions implemented over a 17-year period in a comprehensive stroke center. Among 1250 patients treated with intravenous thrombolysis, 737 were managed during the intervention phase. The proportion of patients receiving thrombolysis within the first hour after hospital arrival increased from 22.5% during the pre-intervention period to 63% between 2015 and 2018 and reached 71% in 2019. Mean door-to-needle time decreased from 58 to 51 min. The most influential interventions included the implementation of a direct-to-CT protocol, healthcare workflow reorganization, and the incorporation of a specialized stroke nurse, each contributing significantly to treatment acceleration (Figure 2).

Similar findings were reported by Davis et al. [20], who retrospectively evaluated factors associated with door-to-needle time among 89 patients receiving alteplase. Hospital arrival mode and the presence of a dedicated stroke nurse were identified as significant determinants of treatment efficiency.

The timing of stroke pathway activation also emerged as a critical factor. Antunes et al. [21] analyzed 226 patients presenting with cerebrovascular disease and demonstrated that activation of the Stroke Code during triage substantially reduced door-to-imaging times. Patients whose stroke pathway was activated during triage underwent computed tomography after a mean of 35 ± 18 min, compared with 38 ± 26 min when activation occurred later and 64 ± 45 min when no activation was performed.

The role of triage assessment tools was explored by Jay et al. [22], who compared the FAST and BEFAST scales in emergency department triage. The implementation of BEFAST significantly increased the proportion of patients assigned to high-priority categories and reduced mean door-to-imaging time from 91.0 to 70.3 min. Patients assessed using BEFAST were more likely to achieve favorable discharge destinations and experienced shorter hospital stays. Although no substantial differences were observed in modified Rankin Scale scores at 90 days, a greater proportion of patients in the BEFAST group achieved lower disability scores.

Overall, the available evidence indicates that treatment times are influenced by multiple interconnected factors, including organizational restructuring, protocol activation, multidisciplinary coordination, specialized stroke personnel, and the implementation of structured triage tools. These interventions generally demonstrated the ability to reduce delays in diagnosis and treatment, although improvements in functional outcomes were not consistently observed across all studies.

#### 3.3.2. Impact of Care Interventions on Clinical Outcomes

Several studies evaluated the effect of structured organizational interventions on neurological recovery, functional outcomes, and treatment success. Li et al. [23] assessed the implementation of a time-based emergency stroke protocol involving interdisciplinary communication pathways, rapid-response teams, and predefined treatment targets. Compared with the control group, the intervention cohort experienced a 12.7% increase in intravenous thrombolysis rates, significant reductions in treatment delays, and improvements in neurological function, motor performance, and stroke-specific quality of life measures.

**Figure 3:**
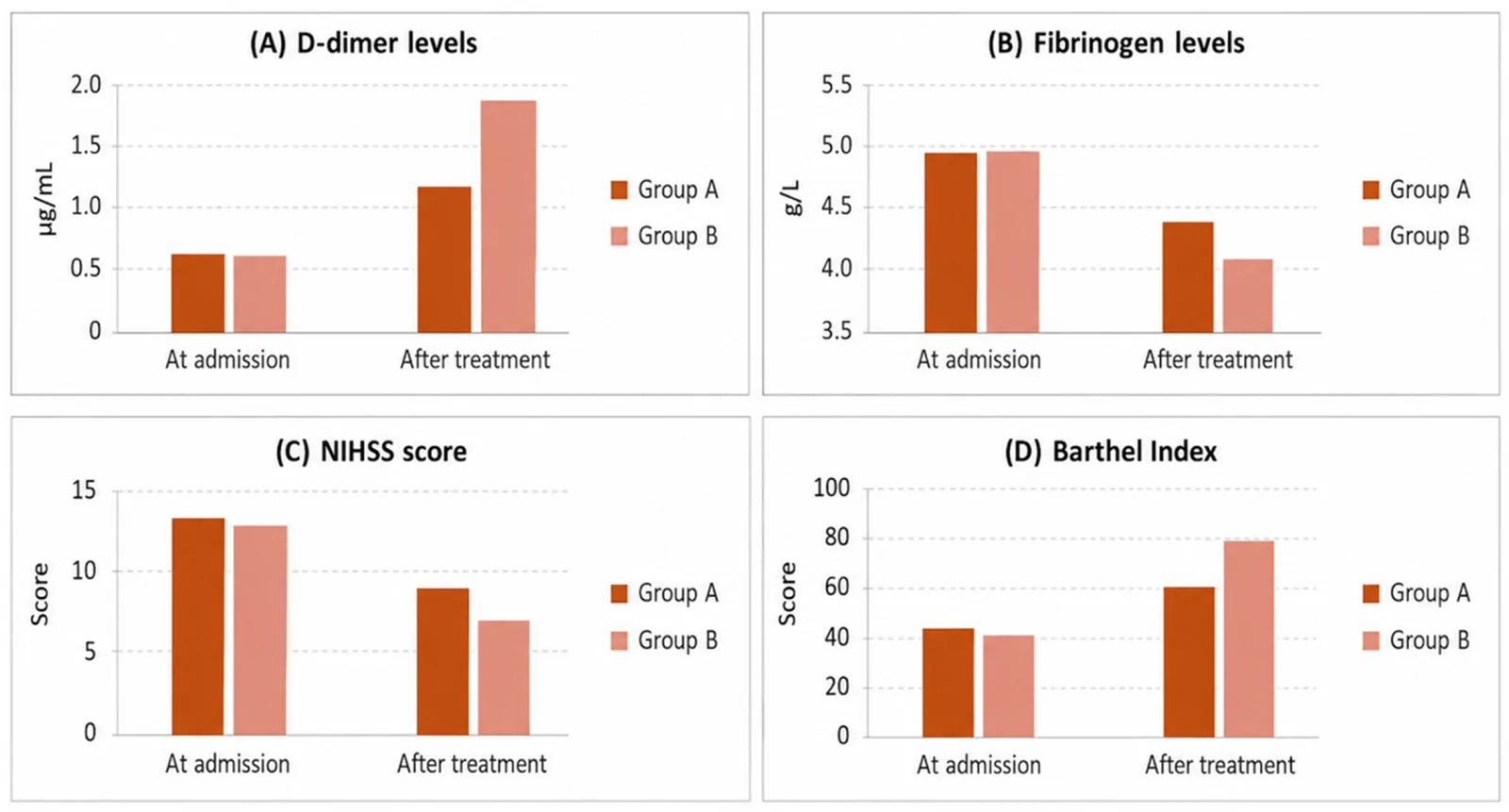
Comparison between groups of coagulation values (**A**,**B**) and functional and neurological recovery (**C**,**D**). Group A represents the control group receiving conventional care, whereas Group B corresponds to the intervention group managed under the structured stroke pathway described by Chen. Source: Prepared by the authors using data from Chen et al. [24].

Integrated prehospital–hospital care models also demonstrated beneficial effects. Chen et al. [24] evaluated an integrated nursing-led stroke pathway and reported significant reductions in admission-to-treatment time and emergency department length of stay. Patients managed within the integrated model exhibited higher thrombolysis success rates and greater improvements in coagulation parameters, neurological status, and functional independence. Furthermore, patient satisfaction was significantly higher, while levels of anxiety and depression were substantially reduced compared with routine care (Figure 3).

Evidence from randomized controlled trials further supported the effectiveness of structured emergency interventions. Xie et al. [25] investigated the impact of enhanced monitoring protocols and simulation-trained healthcare professionals on post-stroke recovery. Although both study groups improved clinically, the intervention group showed greater reductions in neuron-specific enolase and inflammatory biomarkers, including interleukin-6 and tumor necrosis factor-alpha, suggesting a more favorable biological response following stroke.

Similarly, Liu et al. [26] demonstrated that protocolized emergency interventions significantly reduced admission-to-diagnosis and diagnosis-to-treatment intervals. These improvements translated into better outcomes in activities of daily living, quality of life, neurological recovery, and cognitive performance one month after discharge. The intervention group also showed evidence of reduced inflammatory activity and potentially lower risk of post-thrombolysis re-aggregation.

Xue et al. [27] evaluated a structured protocol-based care model focused on early risk assessment, continuous monitoring, and timely implementation of therapeutic interventions. This approach was associated with shorter door-to-needle times, improved neurological and cognitive recovery, and fewer post-thrombolysis complications.

Taken together, these findings suggest that structured emergency care pathways and multidisciplinary collaboration consistently improve process indicators such as treatment times, whereas evidence regarding functional and long-term clinical outcomes remains more heterogeneous.

#### 3.3.3. Contribution of Specialized Stroke Professionals to Care Optimization

**Figure 4:**
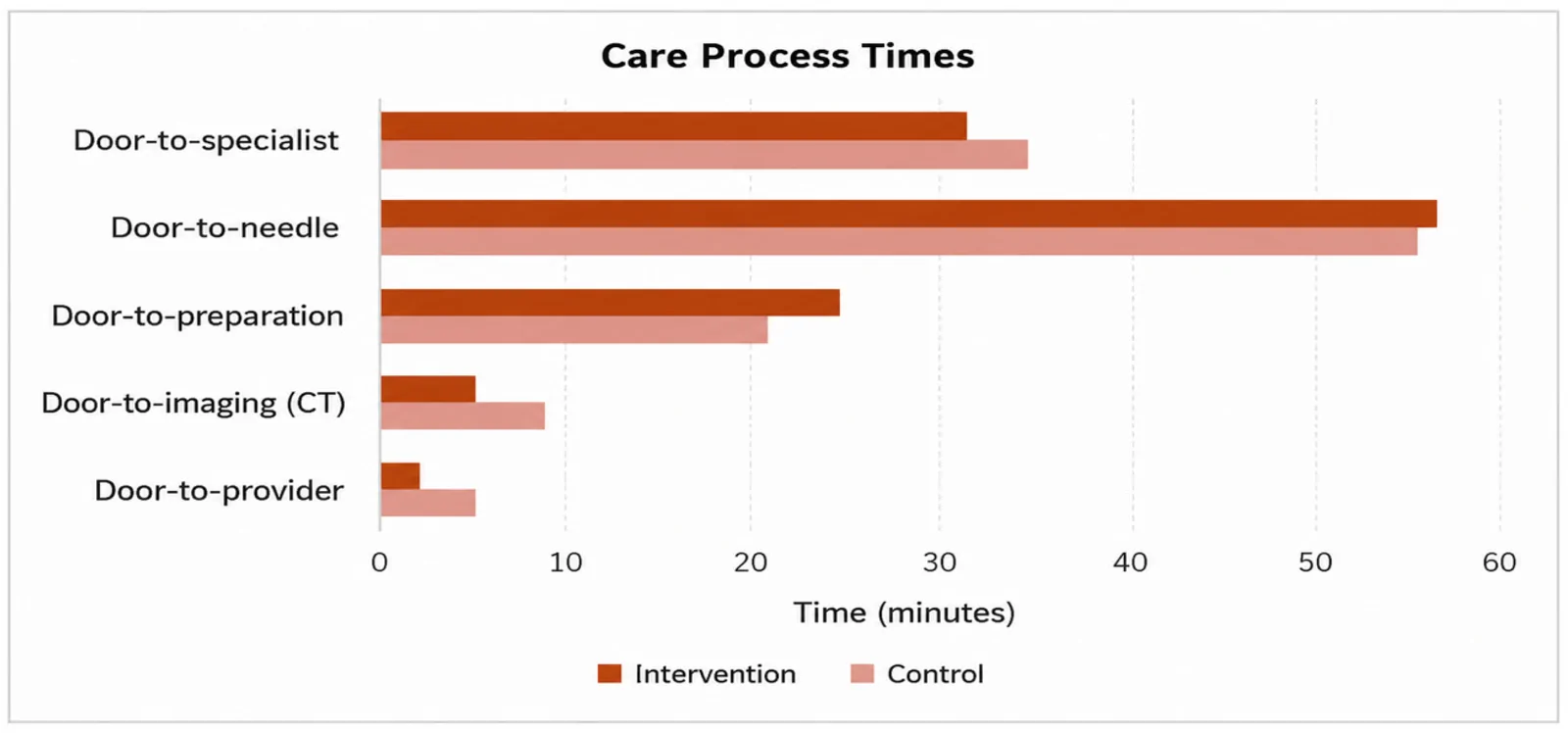
Differences between groups at different care times. Source: Prepared by the authors using data from Olson et al. [28].

Several studies highlighted the contribution of specialized stroke professionals to improving emergency stroke care performance. Olson et al. [28] evaluated a nurse-led telemedicine stroke protocol involving 447 patients. Following implementation, significant reductions were observed in door-to-provider and door-to-computed tomography times. Although preparation time increased, no significant differences were found in door-to-needle or door-to-specialist times (Figure 4).

Integrated care approaches involving both prehospital and in-hospital interventions also demonstrated substantial benefits. Zhan et al. [29] investigated a model combining community education, structured triage procedures, and priority neuroimaging communication pathways. The intervention group showed significantly shorter door-to-needle and door-to-imaging times and achieved a markedly higher proportion of thrombolysis within the first hour after hospital arrival. Furthermore, the intervention independently predicted early neurological improvement and favorable functional outcomes at discharge.

The role of specialized stroke nurses emerged as one of the most consistently reported determinants of improved performance. In a systematic review involving 2393 patients, Kumar et al. [30] demonstrated that the incorporation of stroke nurse specialists was associated with reductions in door-to-needle, door-to-imaging, and symptom-onset-to-treatment times. Moreover, patients managed within stroke nurse-led pathways were significantly more likely to receive intravenous thrombolysis.

Comparable findings were reported by Xu et al. [31], who observed a reduction in mean door-to-needle time from 36 to 25 min following the introduction of a stroke nurse specialist. This intervention was also associated with better functional outcomes at 90 days, although no significant differences were observed in early neurological improvement.

**Figure 5:**
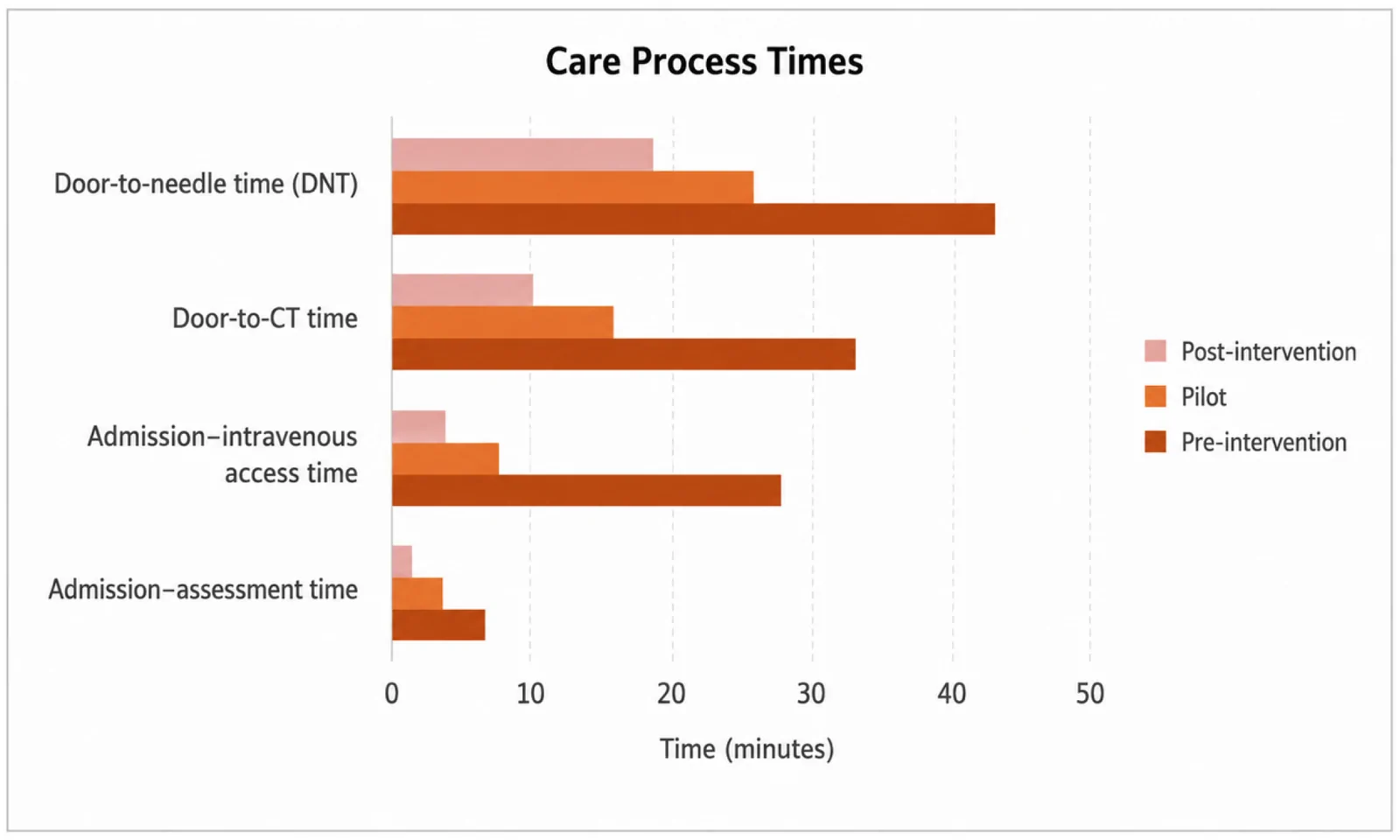
Comparison between the pre-intervention, pilot, and post-intervention groups of the following times: admission-assessment, admission-venous access, admission-CT scan, and door-to-needle (DNT). Source: Prepared by the authors using data from Li et al. [32].

Likewise, Li et al. [32] reported remarkable reductions in admission-to-assessment and door-to-needle times after the incorporation of specialized stroke nurses into emergency department workflows. Door-to-needle time decreased from 43 min during the pre-intervention phase to 20 min following full implementation of the program (Figure 5).

Collectively, these studies indicate that specialized stroke professionals, particularly stroke nurse specialists integrated within multidisciplinary teams, play a pivotal role in optimizing emergency stroke care. Their involvement contributes to faster clinical assessment, more efficient protocol activation, reduced treatment delays, increased reperfusion rates, and improved functional outcomes, reinforcing the value of dedicated stroke expertise within modern emergency care systems.

## 4. Discussion

The findings of this systematic review indicate that the implementation of structured organizational strategies and protocol-driven care pathways contributes substantially to improving the management of acute ischemic stroke in emergency settings. Across the studies included, the most consistent benefits were observed in the reduction in critical treatment intervals, particularly door-to-needle (DNT) and door-to-imaging times, together with increased rates of intravenous thrombolysis and improvements in neurological and functional outcomes. These results reinforce the widely accepted concept that acute stroke care is highly time-dependent and that optimizing healthcare processes can directly influence patient prognosis.

To our knowledge, this review is among the first to comprehensively synthesize the most recent evidence regarding emergency organizational strategies for acute ischemic stroke published after the implementation of the updated PRISMA 2020 recommendations and the widespread adoption of contemporary stroke pathways. Rather than evaluating isolated interventions, the review integrates evidence on multidisciplinary coordination, protocol activation, triage optimization, and specialized stroke professionals, providing a broader perspective on emergency stroke care.

An important finding of the present review is that improvements were consistently observed in organizational performance indicators, particularly door-to-needle and door-to-imaging times. However, improvements in neurological or functional outcomes were less consistent across studies. This discrepancy is not unexpected, since patient outcomes are influenced by multiple factors beyond treatment times, including stroke severity, comorbidities, prehospital delays, and access to reperfusion therapies.

Although no single intervention emerged as universally superior, the available evidence consistently supports a multimodal organizational strategy that combines early stroke recognition, protocolized stroke-code activation, direct access to neuroimaging, multidisciplinary coordination, and the participation of dedicated stroke professionals. Rather than isolated interventions, the effectiveness of acute stroke care appears to depend on the integration of these complementary organizational measures.

One of the most relevant findings emerging from the reviewed literature is the importance of organizational optimization through structured stroke pathways. Park et al. [19] demonstrated that the sustained implementation of quality-improvement measures over time resulted in a progressive increase in the proportion of patients receiving reperfusion therapy within the first hour after hospital arrival. The introduction of a direct-to-CT protocol, workflow reorganization, and the incorporation of a specialized stroke nurse were identified as key contributors to these improvements. These findings suggest that successful stroke care depends not on a single intervention but rather on the synergistic effect of multiple organizational strategies integrated within a coordinated multidisciplinary framework.

Similarly, Li et al. [23] reported that the implementation of time-based clinical pathways supported by interdisciplinary collaboration significantly reduced treatment delays and increased thrombolysis rates. Importantly, these organizational improvements were accompanied by better neurological recovery, functional outcomes, and quality-of-life indicators. Such findings support previous evidence demonstrating that reductions in treatment times are closely associated with improved clinical outcomes and reinforce the need for streamlined stroke pathways within emergency departments.

Telemedicine has emerged as a promising approach for optimizing stroke care, particularly in regions with limited access to specialist services. Olson et al. [28] found that a nurse-led telemedicine protocol improved door-to-provider and door-to-imaging times. However, the absence of significant changes in door-to-needle time and the increase in treatment preparation time suggest that telemedicine interventions must be integrated within broader multidisciplinary workflows to achieve maximum effectiveness. These findings highlight that technological innovations alone may be insufficient unless supported by comprehensive organizational adaptations.

The review also emphasizes the value of integrated care models that combine prehospital and in-hospital interventions. Zhan et al. [29] demonstrated that a strategy incorporating community education, nurse-led triage, and prioritized communication with neuroimaging services significantly reduced both door-to-needle and door-to-imaging times while increasing the proportion of patients treated within recommended therapeutic windows. Likewise, Chen et al. [24] showed that an integrated stroke care model improved treatment efficiency, thrombolysis success rates, and functional recovery while simultaneously reducing emergency department length of stay. Furthermore, improvements in patient and family satisfaction, together with reductions in negative emotional responses, suggest that integrated stroke pathways may positively influence not only clinical outcomes but also patient-centered aspects of care.

Another important finding concerns the early activation of stroke pathways during triage. Antunes et al. [21] demonstrated that Stroke Code activation at the triage stage significantly reduced door-to-imaging times compared with delayed activation or absence of activation. These results underscore the strategic importance of triage as the first point of contact within the emergency care pathway and highlight the need for healthcare professionals capable of rapidly identifying stroke symptoms and initiating appropriate diagnostic and therapeutic processes.

In this context, the use of structured screening tools appears to further enhance early recognition. Jay et al. [22] observed that implementation of the BEFAST scale increased prioritization of stroke patients and reduced door-to-imaging times compared with the traditional FAST scale. These findings may be explained by the inclusion of balance and visual symptoms, which are commonly associated with posterior circulation strokes and may be overlooked using FAST alone. Although no significant differences were observed in functional disability at 90 days, shorter hospital stays and more favorable discharge destinations suggest that earlier recognition may confer meaningful clinical benefits.

One of the most consistent themes across the reviewed studies was the contribution of specialized stroke professionals to improving care delivery. Kumar et al. [30] demonstrated that the incorporation of stroke nurse specialists was associated with shorter door-to-needle, door-to-imaging, and symptom-onset-to-treatment intervals, as well as increased rates of intravenous thrombolysis. These findings were supported by the studies of Davis et al. [20], Xu et al. [31], and Li et al. [32], all of which identified specialized stroke nurses as important facilitators of more efficient care processes and improved patient outcomes. Although the present review adopts a broader multidisciplinary perspective, the evidence consistently indicates that dedicated stroke expertise represents a valuable component of modern stroke systems of care.

Beyond their influence on treatment times, several studies demonstrated that structured interventions can directly affect clinical recovery. Randomized controlled trials conducted by Xie et al. [25], Liu et al. [26], and Xue et al. [27] reported improvements in neurological function, cognitive performance, and functional independence among patients receiving enhanced protocolized care. These interventions were also associated with lower inflammatory biomarker levels and reduced rates of post-thrombolysis complications. Such findings suggest that optimized stroke care pathways may exert benefits beyond process indicators, translating into measurable improvements in patient recovery and long-term prognosis.

Overall, the evidence synthesized in this review supports the implementation of multidisciplinary, protocol-driven stroke pathways that prioritize rapid diagnosis, early treatment, and coordinated communication among healthcare professionals. The observed improvements in treatment efficiency and clinical outcomes indicate that organizational excellence is a fundamental determinant of successful acute ischemic stroke management.

### Limitations and Future Research

Several limitations should be considered when interpreting the findings of this review. First, substantial heterogeneity existed among the included studies regarding design, patient populations, interventions, outcome measures, and healthcare settings. This variability limited direct comparisons between studies and precluded quantitative meta-analysis. Second, some investigations included relatively small sample sizes, potentially reducing statistical power and limiting the generalizability of their findings. Third, long-term follow-up data were unavailable in several studies, making it difficult to determine the sustained impact of organizational interventions on functional recovery, quality of life, and long-term disability.

Another limitation is that many of the included studies were conducted in specific healthcare systems with established stroke networks, which may limit the applicability of findings to settings with different organizational structures or resource availability. Furthermore, while numerous studies identified associations between organizational interventions and improved outcomes, causal relationships remain difficult to establish due to the observational nature of much of the available evidence.

Despite these limitations, the consistency of findings across diverse study designs strengthens confidence in the overall conclusions. The available evidence suggests that structured stroke pathways, optimized emergency workflows, multidisciplinary collaboration, and specialized stroke expertise contribute meaningfully to reducing treatment delays and improving patient outcomes.

Another limitation is that a substantial proportion of the included studies originated from Asian healthcare systems. Although these studies provide valuable evidence regarding organizational strategies, differences in healthcare organization may limit the direct generalizability of some findings to other settings.

Future research should focus on large-scale multicenter studies and pragmatic clinical trials evaluating specific organizational interventions within diverse healthcare environments. Further investigation is also warranted to assess the long-term effects of integrated stroke care pathways on functional recovery, quality of life, healthcare utilization, and cost-effectiveness. Finally, studies exploring the contribution of individual professional roles within multidisciplinary stroke teams may help identify the most effective strategies for optimizing acute ischemic stroke care and maximizing patient outcomes.

## 5. Conclusions

This systematic review demonstrates that the implementation of structured, multidisciplinary, and protocol-driven care strategies significantly contributes to the optimization of acute ischemic stroke management in emergency settings. The available evidence consistently indicates that organizational interventions aimed at streamlining care pathways are associated with improvements in both process indicators and patient outcomes.

The findings suggest that critical treatment intervals, particularly door-to-needle and door-to-imaging times, are influenced by multiple organizational and clinical factors. Early activation of Stroke Code protocols, the use of dedicated triage tools, workflow reorganization, and the integration of specialized multidisciplinary teams were identified as key determinants of treatment efficiency. The implementation of these strategies facilitates faster diagnosis and initiation of reperfusion therapies, which is particularly relevant in a time-dependent condition such as acute ischemic stroke.

Furthermore, the reviewed studies demonstrate that structured care pathways and coordinated multidisciplinary interventions positively influence clinical outcomes. Protocolized management, effective communication between prehospital and hospital services, and evidence-based monitoring and treatment procedures were associated with shorter treatment delays, higher intravenous thrombolysis rates, improved neurological and functional recovery, and a lower incidence of complications. These findings support the growing body of evidence indicating that healthcare system organization is a critical determinant of stroke outcomes.

An additional finding of this review is the relevance of specialized stroke expertise within multidisciplinary teams. Healthcare professionals involved in emergency stroke care contribute substantially to the early recognition of symptoms, rapid activation of stroke pathways, timely administration of reperfusion therapies, and continuous patient monitoring. In particular, the incorporation of specialized stroke nurses was consistently associated with reductions in critical treatment times and improvements in functional outcomes, highlighting the value of dedicated stroke expertise within contemporary stroke systems of care.

The evidence synthesized in this review suggests that protocol-driven multidisciplinary emergency care improves the efficiency of acute ischemic stroke management, particularly by reducing treatment delays. However, the impact on clinical and functional outcomes is less consistent across studies. The findings support the implementation of integrated organizational strategies combining early recognition, rapid imaging, protocol activation, and coordinated multidisciplinary teams, while highlighting the need for additional high-quality multicenter studies to define the most effective care models.

Future multicenter studies with standardized outcome measures are needed to determine which combination of organizational strategies provides the greatest benefit across different healthcare systems.

## Data Availability

No new data were created or analyzed in this study. Data sharing is not applicable to this article.

## Supplementary Materials

The following supporting information can be downloaded at: https://www.mdpi.com/article/10.3390/neurolint18080148/s1, Table S1: PRISMA 2020 Checklist [33].

## Appendix A

**Table A1:** Summary table of the reviewed articles. Source: Prepared by the authors using data extracted from the studies included in the review [19,20,21,22,23,24,25,26,27,28,29,30,31,32].

Authors. Year.	Type of Study.	Objective.	Level of Methodological Quality.
Park PSW, Frost T, Tan S, Wong J, Pope A, Dewey HM, Choi PMC [19]. 2022	Retrospective study	To analyze the evolution of door-to-needle time in a Melbourne stroke center over 17 years and determine the factors that have influenced its reduction.	NOS: 8
Davis NW, Bailey M, Buchwald N, Farooqui A, Khanna A [20]. 2021	Retrospective study	Determine the factors that influence the time elapsed until the administration of thrombolytic treatment.	NOS: 7
Antunes R, Costeira C, Pereira Sousa J, Santos C [21]. 2024	Retrospective study	Analyze the influence of Stroke Code activation on door-to-CT scan time.	NOS: 8
Jay D, Wheatley R, Smith L, Davis KJ [22]. 2024	Retrospective study	Analyze whether the application of the BEFAST scale in triage improves the identification of stroke cases.	NOS: 8
Li YF, Feng GQ, Ao YP, Gu B, Wang J, Xiao AF, You JQ [23]. 2025	Quasi-experimental time cohort study	To evaluate the impact on the care process and clinical prognosis of establishing a new protocol based on care times.	NOS: 9
Chen Y, Mao Y, Chen L [24]. 2023	Retrospective study	Analyze the possible benefit of establishing integrated nursing care on different variables.	NOS: 7
Xie H, Gao M, Lin Y, Yi Y, Liu Y [25]. 2022	Randomized clinical trial	To analyze the effect of monitoring and nursing interventions on cognitive impairment and neurological function in patients with acute stroke versus the effect of routine nursing care.	JADAD: 3
Liu Y, Wang C, Han Y [26]. 2023	Randomized clinical trial	To determine the impact of a new nursing approach to urgent stroke care, along with the use of combination therapy, versus the impact of the standard approach and treatment.	JADAD: 3
Xue L, Deng J, Zhu L, Shen F, Wei J, Wang L, Chen Q, Wang L [27]. 2023	Randomized clinical trial	To evaluate the effectiveness of the predictive nursing model in stroke care, that is, the establishment of action plans for the different phases of care and their possible complications.	JADAD: 3
Olson DM, Provencher M, Stutzman SE, Hynan LS, Novakovic S, Guttikonda S, Figueroa S, Novakovic-White R, Yang JP, Goldberg MP [28]. 2022	Prospective non-randomized study	To evaluate the effects of implementing a nursing protocol in telehealth stroke care.	NOS: 8
Zhan T, Xu C [29]. 2025	Retrospective study	To assess the effectiveness of a series of nursing interventions at different levels of care on stroke care in its acute phase and their impact on recovery.	NOS: 9
Kumar A, Kumar M, Verma P, Pal R, Nagi M, Mahesh KV, Munjal DK, Kaur S, Aggarwal A, Padhi BK, Khurana D [30]. 2025	Systematic review and meta-analysis	To evaluate the impact of the presence of the “stroke nurse” on care times and clinical outcomes.	AMSTAR 2: 13
Xu ZH, Deng QW, Zhai Q, Zhang Q, Wang ZJ, Chen WX, Gu MM, Jiang T, Zhou JS, Zhang YD [31]. 2021	Retrospective study	To determine the effect of incorporating a stroke nurse in a hospital in Nanjing.	NOS: 9
Li D, Zhang H, Lu X, Zhang L, Liu J [32]. 2022	Retrospective study	To explore the practical effect of an integrated treatment process carried out by the emergency department nursing staff of a hospital.	NOS: 8

## Appendix E

**Table A2:** Comparison between different studies of the variables door-to-needle time, door-to-CT time, and thrombolysis rate. Where two groups existed, the values corresponding to the intervention group were selected. Source: Prepared by the authors using data extracted from the studies included in the review [19,20,21,22,23,24,25,26,27,28,29,30,31,32].

Study	Door-to-Needle Time (Median (IQR), Mean ± SD (Min–Max) or Mean ± SD)	Door-to-CT Time (Median (IQR), Mean ± SD (Min–Max) or Mean ± SD)	Thrombolysis Rate (%)
Park et al. [19]	54 (40–68)	14 (8–24)	
Davis et al. [20]	53.74 ± 38.06 (7–233)		
Antunes et al. [21]		50 ± 37	
Jay et al. [22]		70.3 ± 63.4 (4–374)	7.2% * * treatment not available at the center until 2021
Li et al. [23]	38.16 ± 2.12		57.6%
Chen et al. [24]	59.92 ± 11.73		55.38%
Xie et al. [25]			
Liu et al. [26]			
Xue et al. [27]	36.57 ±8.63 * * visual estimation of the graph provided by the authors		
Olson et al. [28]	56 (25.0)	5 (11.0)	
Zhan et al. [29]	39.7 ± 8.6	18.6 ± 5.3	
Kumar et al. [30]			
Xu et al. [31]	25(17–35)		
Li et al. [32]	20 ± 6 * * visual estimation of the graph provided by the authors		
